# Lumbar Spondylolysis After Posterior Corrective Fusion for Adolescent Idiopathic Scoliosis: A Report of Two Cases and Literature Review

**DOI:** 10.7759/cureus.76854

**Published:** 2025-01-03

**Authors:** Takuya Nikaido, Yohei Matsuo, Kazuyuki Watanabe, Kinshi Kato, Hiroshi Kobayashi, Masataka Nakamura, Takuya Kameda, Yoichi Kaneuchi, Michiyuki Hakozaki, Miho Sekiguchi, Koji Otani, Shoji Yabuki, Yoshihiro Matsumoto

**Affiliations:** 1 Department of Orthopaedic Surgery, Fukushima Medical University School of Medicine, Fukushima, JPN; 2 Department of Orthopaedic Surgery, Fukushima General Health and Welfare Centre, Fukushima, JPN; 3 Department of Research for Spine and Spinal Surgery, Fukushima Medical University, Fukushima, JPN; 4 Department of Orthopaedic Surgery, Fukushima Medical University, Fukushima, JPN; 5 Higashi-Shirakawa Orthopaedic Academy, Fukushima Medical University School of Medicine, Fukushima, JPN

**Keywords:** adjacent segment disease (asd), adolescent idiopathic scoliosis (ais), badminton, long spinal fusion, lumbar spondylolysis

## Abstract

After corrective surgery for adolescent idiopathic scoliosis (AIS), patients can return to sports activities without restrictions. While there have been many reports of long-term disc degeneration between adjacent segments after posterior corrective fusion, the effects of sports activities on adjacent segments after corrective fusion surgery are not well understood. Particularly, cases of acquired spondylolysis after long fusion surgeries for scoliosis are rare. In this report, we present two cases of AIS in which patients continued to play badminton at a high-performance level following posterior corrective fusion surgery and developed lumbar spondylolysis in the lower instrumented adjacent vertebrae. We also discuss the factors that should be considered when returning to sports after corrective posterior fusion surgery for AIS.

## Introduction

Returning to sports after corrective surgery is a significant consideration in patients with adolescent idiopathic scoliosis (AIS). Despite the absence of standardized guidelines, current evidence indicates that patients can safely return to various sports, including those that require extreme spinal movements, without serious complications [1,2]. On the other hand, there are reports that only 60% of AIS patients are able to return to sports at an equal or higher level of physical activity than before surgery; therefore, the actual situation is not clear [3]. Adjacent segment disease (ASD) is a common complication in patients with long-term outcomes following spinal fusion surgery for AIS, with an incidence rate of approximately 26-32% [4,5]. However, the new-onset spondylolysis after corrective fusion surgery for AIS is extremely rare [6-8].

We report two cases of spondylolysis occurring in the caudal adjacent vertebrae of the lower instrumented vertebra (LIV) after posterior corrective fusion surgery for AIS (a 13-year-old girl and a 12-year-old girl). In both cases, no bony healing of the spondylolysis was observed. Here, we share our experience and consider the relationship between sports activity and lumbar spondylolysis after posterior corrective fusion surgery for AIS.

## Case presentation

Case 1 first visited our institution in October 2019, and case 2 first visited our institution in February 2019; therefore, these cases were consecutive.

Case 1

**Figure 1:**
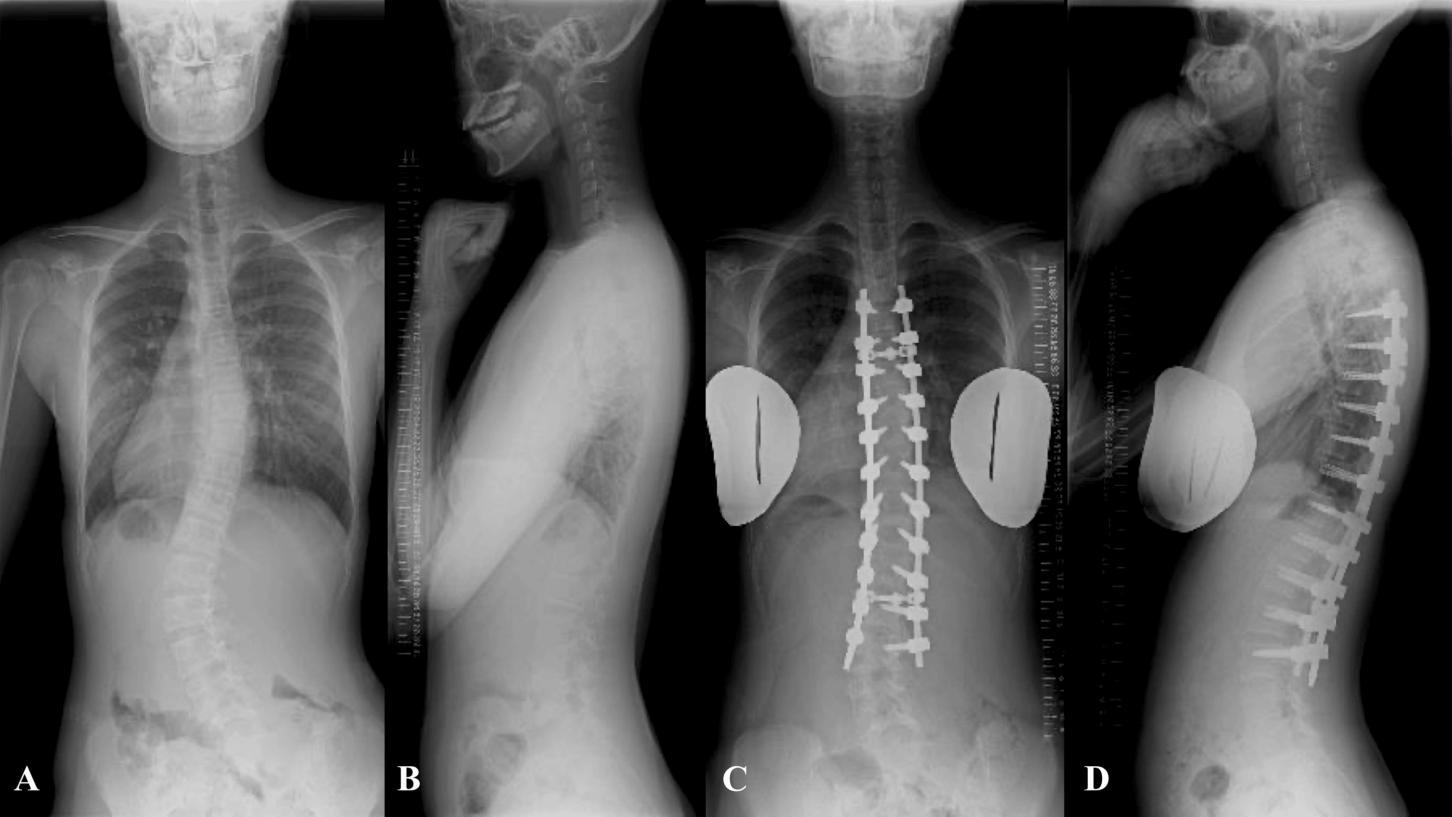
Case 1. Plain radiographs of the whole spine in a standing position. A: preoperative AP view, B: preoperative lateral view, C: postoperative AP view, and D: postoperative lateral view. AP: anteroposterior

A 13-year-old girl was referred to our hospital with a chief complaint of right back and left lumbar prominence. She had no back pain or lower limb symptoms, and all neurological examinations were normal. There were no significant findings related to her growth or medical history, and she was a member of a swimming club in junior high school. Plain radiographs of the standing whole spine showed a 40° structural curve from T11 to L4 (32° in left side bending position) and a 31° non-structural curve from T5 to T11 (21° in right side bending position) (Figures 1A, 1B). Computed tomography (CT) revealed no developmental abnormalities in the spinal structure, including lumbar spondylolysis. Therefore, the patient was diagnosed with AIS based on Lenke classification type 5C. She had not reached menarche, and Risser grade 2. Four months later, after orthotic bracing, plain radiographs of the whole spine showed that the T11 to L4 curve had progressed to 47°, and surgery was planned (Figures 1A, 1B). The spino-pelvic parameters were the following: Pelvic incidence (PI): 65°, Pelvic tilt (PT): 19°, Sacral slope (SS): 44°, and Lumbar lordosis (LL): 55°.

**Figure 2:**
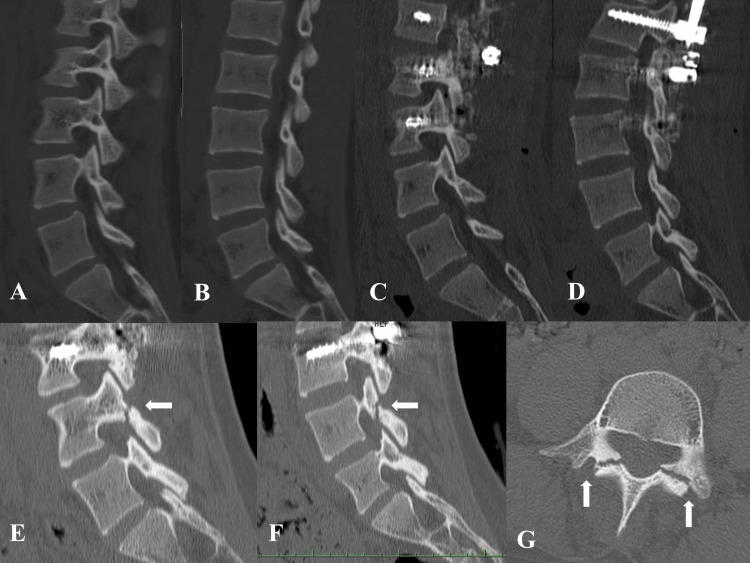
Case 1. Sagittal and axial CT images for the PI of L4. Preoperative and one week after surgery CT scans show no abnormalities in the pars interarticularis (PI) of L4 (A: preoperative right PI, B: preoperative left PI, C: right PI one week after surgery, and D: left PI one week after surgery). CT at the onset of low back pain, 43 months postoperatively, shows bilateral spondylolysis of L4 (E: right PI, F: left PI, and G: L4 axial). PI: pars interarticularis

Nine months after diagnosis, posterior corrective fusion surgery was performed from T5 to L3 (Figures 1C, 1D). All dorsal laminae in the corrective fusion range were decorticated, and local bone obtained from the excision of the spinous processes and facet joints was grafted. The main thoracic curve at T5-T11 was non-structural, but the right-back prominence and low left shoulder were also the main complaints and were therefore deemed to be within the range of correction. The major lumbar curve from T11 to T4 was corrected to 9°, and the main thoracic curve from T5 to T11 to 5°. Postoperative spino-pelvic parameters were 66° for PI, 18° for PT, 46° for SS, and 67° for LL, indicating augmentation of lumbar lordosis. Preoperative and one week after surgery, CT scans show no abnormalities in the pars interarticularis (PI) of L4 (Figures 2A-2D).

The postoperative course was uneventful, and the patient did not complain of lower back pain. She was instructed to wear a trunk orthosis for three months and to refrain from sports for six months after surgery. The patient was allowed to play sports from the seventh month after surgery, and she returned to swimming. Twenty-four months after the surgery, she joined her high school’s badminton club. She followed a routine of practicing for more than three hours on weekdays and participating in matches and tournaments on weekends. At 43 months after the operation (at age 17 years), she experienced severe lower back pain during a badminton match, which made it difficult for her to continue playing. She presented to our hospital, where CT revealed bilateral spondylolysis at the L4 (Figures 2E-2G). She was treated conservatively with cessation of sports activity, lumbosacral orthosis, and stretching was introduced to alleviate hamstring tightness. After three months of conservative therapy, CT was performed five months later to assess bony healing. Although bony healing of spondylolysis was not observed, the patient strongly desired a return to badminton competition. After returning to competition, she did not complain of lower back pain and could perform exercises at the pre-injury level. Twelve months after the onset of lumbar spondylolysis, the patient remained free of low back pain. We plan to conduct regular follow-up in the future.

Case 2

**Figure 3:**
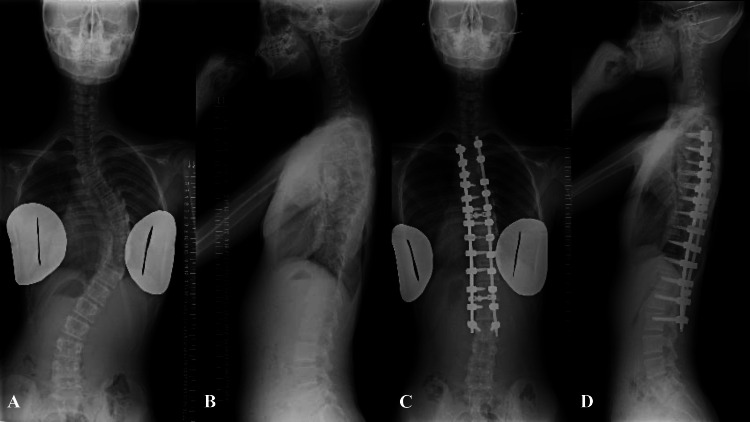
Case 2. Plain radiographs of the whole spine in a standing position. A: preoperative AP view, B: preoperative lateral view, C: postoperative AP view, and D: postoperative lateral view.

A 12-year-old girl visited a doctor with a chief complaint of shoulder-height asymmetry. She had no back pain or lower limb symptoms, and all neurological examinations were normal. There were no significant findings in her growth or medical history, and she had not yet begun menstruation. She had been a badminton club member since the age of 11 years. Plain radiographs of the whole spine showed a 50° structural curve at T5 to T12 (47° in right side bending position) and a 27° structural curve at T1 to T5 (26° in left side bending position), and her condition was diagnosed as AIS of Lenke classification type 2 with Risser grade 0. Five months later, after orthotic bracing, plain radiographs of the whole spine showed that the T5 to T12 curve had progressed to 64°, and surgery was planned (Figures 3A, 3B). The spino-pelvic parameters were the following: PI: 56°, PT: 14°, SS: 42°, and LL: 46°.

**Figure 4:**
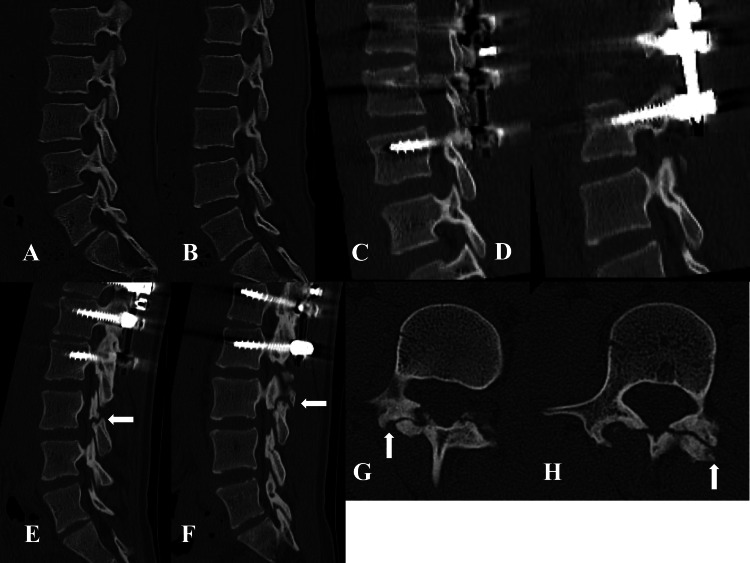
Case 2. Pre- and postoperative sagittal and axial CT images for the PI of L3. Preoperative and one week after surgery CT scans show no abnormalities in the PI of L3 (A: preoperative right PI, B: preoperative left PI, C: right PI one week after surgery, and D: left PI one week after surgery). At a regular check-up, 50 months postoperatively, bilateral L3 spondylolysis was observed (E: sagittal view of right PI, F: sagittal view of left PI, G: axial view of right PI, and H: axial view of left PI). PI: pars interarticularis

Ten months after diagnosis, posterior correction and fusion surgery were performed from T3 to L2 (Figures 3C, 3D). All dorsal lamina in the corrective range were decorticated, and local autogenous bone was grafted. The major thoracic curve from T5 to T12 was corrected to 9°, and the proximal thoracic curve from T1 to T5 to 15°. Postoperative spino-pelvic parameters were 58° for PI, 15° for PT, 43° for SS, and 51° for LL, indicating augmentation of lumbar lordosis. Preoperative and one week after surgery, CT scans show no abnormalities in the PI of L3 (Figures 4A-4D).

The patient reported no postoperative back pain. She was instructed to wear a trunk orthosis for three months and to refrain from sports for six months after surgery. At the six-month follow-up examination, the patient complained of mild back pain; however, the radiographic examination revealed no obvious abnormalities. She was allowed to engage in sports from the seventh month after surgery. She joined a badminton club in junior high school and began to play. She occasionally experienced back pain that did not interfere with her daily life or sports activities. At a regular checkup 26 months after the surgery, the patient did not complain of back pain. She entered high school 27 months after the surgery and joined a badminton club. Compared with junior high school, the extent of badminton practice increased significantly, and the number of matches played during the weekend also increased. She practiced for three hours every weekday and played in matches every weekend. She experienced severe lower back pain immediately after a match 42 months after the operation (at age 17 years) and had difficulty moving.

The patient was treated with conservative therapy, such as medication and local injections, at a local clinic. As her lower back pain eased, she continued playing badminton without undergoing a thorough examination. At a regular checkup 50 months after surgery, plain radiography revealed suspected L3 spondylolysis. CT was performed, and the patient was diagnosed with bilateral L3 end-stage spondylolysis (Figures 4E-4H). As terminal spondylolysis was present, we did not expect bony healing to occur, and as the lower back pain resolved, no active treatment was performed. There were no further complaints of lower back pain, and the patient could resume badminton throughout high school. She retired from the sport after graduating from high school. The patient was scheduled for regular follow-ups.

## Discussion

Idiopathic scoliosis and spondylolysis in adolescence

A significant number of patients with AIS have concomitant spondylolysis, with a prevalence of 7%. [9]. However, to the best of our knowledge, there have been only three reports of new-onset spondylolysis or spondylolisthesis occurring after corrective surgery for scoliosis [6-8]. Based on these reports, the two cases reported here are considered extremely rare. Table 1 shows a comparison of the background and course of development of lumbar spondylolysis in previous reports and the present cases.

**Table 1:** Characteristics of reported cases of spondylolysis or spondylolisthesis after corrective spinal fusion for AIS. UIV: Upper instrumented vertebra, LIV: Lower instrumented vertebra

Author, Year	Sex	Pathophysiology	Age at scoliosis surgery (years)	Type of surgery	UIV	LIV	Age at spondylosis or spondylolisthesis onset (years)	Spondylolysis or Spondylolishtesis	Sports activity
Tietjen R et al. 1976 [6]	F	congenital	11	two stage scoliosis fusion	T3	L4	23	L5 Spondylolishtesis (Grade I)	N/A
Friedman R et al. 1984 [7]	M	idiopathic	11	posterior spinal fusion with Harrington instrumentation	N/A	L5	14	L5 Spondylolishtesis (Grade II)	hockey
Huang Y et al. 2024 [8]	F	idiopathic	17	long spinal fusion after growing rod implantation and rod extension	T3	L4	20	L5 spondylolysis with L4-S1 instability	N/A
Our report: Case 1	F	idiopathic	14	posterior corrective spinal fusion	T5	L3	17	L4 spondylolysis	badminton
Our report: Case 2	F	idiopathic	13	posterior corrective spinal fusion	T3	L2	17	L3 spondylolysis	badminton

In all previous cases, the LIV was L4 or L5, and spondylolysis occurred in L5 vertebrae. All of these cases were discussed as ASD caused by mechanical stress. However, since the L5 vertebrae are also commonly affected in ordinary spondylolysis and spondylolysis that occurs concomitantly with AIS, it is possible that there was a contribution from the anatomical characteristics of L5. In contrast, in the present cases, the injured vertebrae were L3 and L4, which were adjacent to the LIV, and the same mechanism as that of ASD after spinal fusion surgery was considered.

Return to sport after surgery for AIS and ASD

AIS is the most common form of scoliosis, affecting approximately 2-4% of adolescents [10-13]. Of these, approximately 10% require some form of treatment, and up to 0.1% undergo surgery [14]. The goal of treatment is to prevent curve progression and improve cosmetic and functional outcomes. Surgical treatment of AIS has evolved significantly over the past two decades. Between 1997 and 2012, posterior fusion became the mainstay of treatment, with the rate of posterior fusion increasing from 63.4% to 94.1% [15]. Recent studies have shown that adolescents can safely return to sports after surgery for AIS, and the use of modern instrumentation has enabled earlier resumption of activities [1]. Most surgeons allow non-contact sports three months postoperatively, contact sports after six months, and collision sports after 12 months [1]. However, a few reports suggest that the average time to return to sports is approximately eight months [16]. Furthermore, there are also studies reporting a range of 6-18 months for return to sports activities [2]. Factors affecting early return include being younger, having a higher Lenke type, and having lower main curve severity [17]. Although many patients can resume their preoperative activities, some move on to low-impact sports because of reduced spinal mobility and flexibility [2,16]. Despite the lack of established guidelines, current evidence suggests that patients can safely return to various sports, including those that require extreme spinal motion, without experiencing serious complications [1,2].

The incidence of ASD following spinal fusion surgery for AIS is approximately 26-32%. [3,4]. The risk factors for ASD include a long follow-up period, fusion of 10 or more segments, and the extent of fusion, with the highest prevalence in East Asia [18]. Age at the time of surgery and pre-existing disc degeneration were also significant risk factors [19]. Most previous reports on ASD discussed disc degeneration in long-term postoperative cases, and reports on spondylolysis of the adjacent vertebrae are extremely rare [8].

Characteristics of the presented cases and learned precept

Lumbar spondylolysis is a common condition in adolescent athletes, and its prevalence varies depending on the sport. The risk of spondylolysis differed by sex, with baseball, soccer, and hockey having the highest prevalence in males and gymnastics, marching band, and softball for female athletes [20]. Although there are no reports that badminton is a risk factor for spondylolysis, it is an overhead sport that involves repeated extension and rotation of the lumbar spine, and it is thought to be a risk factor for acute and overuse disorders of the lower back in young elite players. Recent biomechanical research has shown that thoracic flexibility affects the stress on the lumbar intervertebral discs and PI. In other words, it is possible that in our cases, the decrease in thoracic flexibility due to spinal fusion increased the mechanical stress on the PI, leading to spondylolysis [21]. In these two cases, the presence of a horizontal fracture line on the bilateral PI suggests that the cause of the spondylolysis was the stress of overextension rather than the rotational stress of the lumbar spine [22]. In particular, in Case 1, the main thoracic curve was non-structural, which may have prevented the development of spondylolysis by reducing the thoracic fusion level and preserving thoracic mobility as much as possible. Furthermore, in both cases, a comparison of preoperative and postoperative spinopelvic parameters showed an increase in LL postoperatively. The increased LL is thought to be one of the causes of spondylolysis. The two cases we encountered were patients with AIS who had undergone posterior corrective fusion surgery and participated in high-level badminton competitions as members of organized teams. In addition to practicing for more than three hours on weekdays, they were exposed to high physical loads due to their participation in weekend matches and championships. Patients such as these commonly develop spondylolysis in the caudal adjacent vertebrae of the LIV. To prevent similar outcomes in the future, preserving the maximum possible number of mobile segments during corrective fusion should be considered in patients who strongly desire to return to high-level competitive sports. Particularly, the LIV should be set more cranially to preserve the mobile segment [23]. In addition, because spinal flexibility decreases following surgery, transitioning to low-impact sports that do not involve rotational forces or excessive lumbar extension should be considered. Improving lower limb muscle tightness and strengthening the trunk muscles, especially the lower spinal extensor, are useful for postoperative AIS patients to return to sports [24,25]. If lower back pain occurs during sports, it is important to visit a medical institution at the earliest for a thorough examination. It is especially advisable to consult a physician who performs scoliosis surgery. If a physician does not understand the pathophysiology, the diagnosis may be delayed. Although ASD is not an uncommon condition, it does not necessarily lead to clinical symptoms [18]. In other words, long-term follow-up and regular screening are essential for the early detection and management of ASD, including spondylolysis.

## Conclusions

In this report, we present two cases of lumbar spondylolysis that occurred after corrective fusion surgery for AIS. Both patients participated in high-level badminton competitions postoperatively. Returning to sports after AIS remains a significant challenge, as high-level sports activities increase mechanical stress on the fixed adjacent segment, increasing the risk of developing lumbar spondylolysis. Sports activities involving rotational force or excessive lumbar extension should be approached cautiously. If a patient develops severe back pain after corrective fusion surgery for AIS, an early diagnosis by a specialist is necessary.
